# Archiving the COVID-19 pandemic in Mass Observation and Middletown

**DOI:** 10.1177/09526951231152139

**Published:** 2023-04-30

**Authors:** Nick Clarke, Clive Barnett

**Affiliations:** 7423 University of Southampton, UK; University of Exeter, UK

**Keywords:** archive, COVID-19 pandemic, diaries, Mass Observation, Middletown

## Abstract

The COVID-19 pandemic generated debates about how pandemics should be known. There was much discussion of what role the human sciences could play in knowing – and governing – the pandemic. In this article, we focus on attempts to know the pandemic through diaries, other biographical writing, and related forms like mass photography. In particular, we focus on the archiving of such forms by Mass Observation in the UK and the Everyday Life in Middletown (EDLM) project in the USA, and initial analyses of such material by scholars from across the human sciences. Our main argument is that archiving the pandemic was informed by, and needs viewing through, the history of the human sciences – including the distinctive histories and human sciences of Mass Observation and Middletown. The article finishes by introducing a Special Section that engages with archiving the pandemic in two senses: the archiving of diaries and related forms by Mass Observation and the EDLM project, and the archiving of initial encounters between researchers and this material by *History of the Human Sciences*. The Special Section seeks to know the pandemic from the human sciences in the present and to archive knowing the pandemic from the human sciences for the future.

## Ways of knowing pandemics

There are many ways of knowing pandemics. During the COVID-19 pandemic, the dominant ways of knowing were the health sciences, especially public health, epidemiology, and virology. But ways of knowing from the human sciences were prominent too. In the UK, where the authors of this introduction were based for the duration of the pandemic, psychologists, behavioural scientists, anthropologists, and historians were included in SAGE (the Scientific Advisory Group for Emergencies), or at least its expert groups (e.g. SPI-B – the Scientific Pandemic Influenza Group on Behaviour). The Government Office for Science asked the British Academy to gather evidence from across the humanities and social sciences on the economic, social, and cultural challenges presented by the pandemic (British Academy, 2021). Still, while the human sciences were included, there were concerns about *how* they were included. The focus of expert groups like SPI-B was primarily on providing advice regarding specific government interventions and how to ensure their effectiveness. There were fewer opportunities for scholars from the human sciences to advise on the assumptions in epidemiological models or to provide alternative views to the ‘population’ or ‘biopolitical’ view of these models; to provide views ‘from the ground’ (Hinchliffe, 2020).

Indeed, these concerns were not just raised by scholars from the human sciences. Richard Horton, editor-in-chief of the *Lancet*, wrote of the ‘handbook’ for dealing with COVID-19 to be found in history and literature – in texts like Defoe's (2003[1722]) *A Journal of the Plague Year*, written to prompt and guide action for when the Marseilles plague of 1720 arrived in England – if only governments and scientists were prepared to look (Horton, 2021). Reflecting on the first year of the COVID-19 pandemic and the UK's response, he called for a better regime of science policymaking based on inclusion of a wider range of specialists and ways of describing and reporting (ibid.). His concern was that statistics on deaths and infections had ‘biologised’ and ‘dehumanised’ the pandemic, stripping it of the biographies of those who died and lived with COVID-19, and so of the meaning and understanding necessary for good policymaking.

Nevertheless, scholars from certain of the human sciences were especially prominent in raising these concerns. When information about the virus first reached the UK, psychologists and associated behavioural scientists were quick to produce editorials, commentaries, reviews, and blogs on the lessons for policymakers from existing theory, experiments, and observation of previous pandemics (e.g. Bonnell *et al*., 2020; Eaton and Kalichman, 2020; Michie *et al*., 2020; Van Bavel *et al*., 2020; Webster *et al*., 2020). As time went by, some psychologists became increasingly critical of the behavioural science apparently influencing the UK's response. These psychologists identify as the ‘social identity tradition’ (Jetten *et al*., 2020). They expected that people would conform with stay-home orders, wear face coverings, get themselves tested, and so on, so long as they were encouraged by ‘identity-leaders’ to feel part of a group, to care about fellow in-group members, to follow group norms; and also if they were facilitated to do so by provision of practical support and opportunities (Drury *et al*., 2021; Drury, Reicher, and Stott, 2020; Elcheroth and Drury, 2020; Reicher and Stott, 2020). These psychologists identified against what they termed ‘the frailty tradition’, which non-psychologists often encounter through behavioural economics with its focus on bias and errors. In this alternative tradition, people are viewed as mentally frail, unable to deal with crises, easily panicked, and best governed through paternalism. It was this frailty tradition that appeared to be influencing the UK's response in early 2020. For example, the concept of ‘pandemic fatigue’ was used to delay the first lockdown of spring 2020 and the so-called ‘circuit breaker’ lockdown of autumn 2020, and to justify the loosening of restrictions over Christmas 2020 (Reicher and Drury, 2021). Yet, for psychologists in the social identity tradition, there is little evidence for pandemic fatigue in the psychology literature, and there was little evidence of waning support for regulations or waning adherence to regulations in the data (ibid.).

If psychologists constituted one vocal group in these discussions, then another group was made up of anthropologists. Some anthropologists working in the UK in early 2020 had significant expertise in pandemic response, or at least epidemic response. They had worked on the recent Ebola outbreaks in West Africa. They called for more anthropology, and more humanities and social science research in general, in the response to COVID-19 – by which they meant more representation on SAGE and its expert groups, a more deliberative process of sourcing and sifting diverse perspectives and evidence, and, at the very least, more use of the human sciences to inform the assumptions and parameters of epidemiological models (Leach, 2020; MacGregor *et al*., 2020). Later, in 2021, their case was re-articulated by Gillian Tett, editor-at-large of the *Financial Times* and a trained anthropologist (Tett, 2021). She compared the ‘anthro-vision’ of anthropology to the ‘tunnel vision’ of polls and models – the dominant ways of knowing the COVID-19 pandemic. She recalled the contributions of anthropologists during the Ebola crisis of 2014, including the lesson that people confront information with culture – ideas of what is proper, local belief systems, ideas of what counts as respectful behaviour, practical realities on the ground, relationships of trust – so there cannot just be one global health script.

These debates about ways of knowing pandemics provide one context for this Special Section. In what follows, we focus on one particular way of knowing pandemics: diaries, biographical writing more broadly, and related forms like mass photography. Diaries and journals were a prominent way of knowing pandemics until the germ theory of Koch and Pasteur in the early 20th century, and the virology that followed advances in microscopy around the middle of the 20th century (Spinney, 2017). We have already mentioned perhaps the most celebrated example of such accounts: Defoe's *A Journal of the Plague Year*. Published in 1722, this journal describes the Great Plague of London in 1665 using a narrator (‘H. F.’) and a combination of facts – statistics from records, pamphlets, medical treatises, and Bills of Mortality (Wall, 2003) – but also stories or ‘human-interest anecdotes’ (Burgess, 2003). Another celebrated example, published well into the 20th century (1947), respected equally as a realistic account of a plague and an allegory of the German occupation of France during the Second World War (Judt, 2020), is Camus’ *The Plague*. Again, there is a narrator, Dr Bernard Rieux, who chronicles events, acts as a historian, and says ‘this happened’ by drawing on his own testimony, that of others, and some written texts – especially the notebooks of another character, Jean Tarrou, in which were compiled ‘a mass of minor details’, ‘conversations overheard in trams or on the street’, and even ‘a minute description of one day in the plague-ridden town’ (Camus, 2020[1947]: 21, 91). We will return to ‘overheards’ and ‘day-surveys’ in our discussion of Mass Observation.

In the 21st century, with so much invested in the health sciences, diaries may no longer be quite so prominent as a way of knowing pandemics. Nevertheless, the COVID-19 pandemic seems to have been a ‘diarological moment’ (Murray *et al*., 2020). From early in the pandemic, people and organisations across the world appear to have written, solicited, and archived journals and other biographical writing. A quick Internet search reveals the pandemic blogs of individuals, series of pandemic diaries run by companies on their websites (often featuring employees), series of pandemic diaries run by media organisations (often featuring healthcare workers), and collections of pandemic diaries built by archives (often local archives with similar collections from previous moments of catastrophe like the Spanish Flu or the Second World War). Also revealed are numerous research projects collecting or using pandemic diaries. In the UK, Michael Ward at Swansea University collected ‘CoronaDiaries’, the Young Foundation collected ‘Covid and Me Diaries’, BritainThinks collected ‘Coronavirus Diaries’ (to supplement its regular opinion polls), geographers in London and Liverpool collected ‘Stay Home Stories’ (to be archived by museums in London and Liverpool), and a multidisciplinary team at University of Edinburgh ran ‘The Lothian Diary Project’ (collecting audio and video diaries, to be archived by Museum and Galleries Edinburgh). In the USA, historians at Arizona State University collected diaries for ‘A Journal of the Plague Year: An Archive of Covid-19’, the Columbia Interdisciplinary Centre for Innovative Theory and Empirics collected diaries alongside video interviews and survey responses for ‘The NYC Covid-19 Oral History, Narrative, and Memory Archive’, and anthropologists at Connecticut and Brown collected journal entries for ‘The Pandemic Journaling Project’. No doubt there are many examples we’ve missed just in these two countries. There will be many more examples in other countries around the world.

The articles in this Special Section are drawn from a seminar series that took as its starting point one of the larger archives of biographical writing about everyday life in the COVID-19 pandemic (at least in the UK): Mass Observation's COVID-19 collections. During the pandemic, the Mass Observation Archive (MOA), based at the University of Sussex in Brighton, collected ‘day diaries’ from people across the UK on 12 May 2020 (*c.*5000 diaries), 2021 (*c.*3000 diaries), and 2022 (*c.*300 diaries); and ‘directive responses’ from its panel of around 600 volunteer writers, who write about suggested topics – outlined in Mass Observation ‘directives’ – three times per year, often in diary form. The MOA also archived diaries and similar writing collected by other organisations and projects during the pandemic, including: Paperchains, which collected writing from prisoners, homeless people, people living with addiction, Armed Forces personnel, young people, and other marginalised groups; and U3A, which collected over 1000 pieces of biographical writing from over 300 older people. The seminar series focused on the methodological challenges presented by these and similar collections for scholars keen to understand and inform responses to the COVID-19 pandemic and future emergencies. Seminar papers can be viewed as a playlist on Mass Observation's YouTube channel (Mass Observation, n.d.). Papers situated the collections in the history of Mass Observation – the original Mass-Observation established in 1937 (with a hyphen) and the MOA established in 1975 – and in relation to other diary projects focused on past pandemics and the COVID-19 pandemic. They considered approaches to the collections from the arts, humanities, and social sciences, including questions of archiving, sampling, and the aesthetics of representation. They asked what the collections can tell us about popular responses to COVID-19, including everyday life during the pandemic. The articles in this Special Section build on some of these seminar papers. Three of them use and reflect on using diaries and other texts, including photographs, archived by the MOA. The fourth article uses diaristic and life writing collected by the Everyday Life in Middletown (EDLM) project – a project based in Muncie, Indiana, inspired by the original Middletown studies of the 1920s and 1930s, the original Mass-Observation, and the MOA. This fourth article helps to extend the Special Section across the Atlantic to the USA, and also to extend this historical relationship between Mass Observation and Middletown.

## Mass-Observation and the human sciences

The original Mass-Observation – an independent research organisation that existed from 1937 to 1949 (not to be confused with Mass-Observation Ltd, a private market research company born out of the original Mass-Observation in 1949, or ‘M-O (UK) Ltd’, a private market research company born out of Mass-Observation Ltd in 1970) – took influences from across the Atlantic in the form of the original Middletown studies, focused on the everyday life of America's new middle class (Hubble, 2010), and the Chicago School of Sociology, focused on the bottom-up sociological study of ordinary people (Campsie, 2016). It can also be situated in broad intellectual developments of the time from across Europe: the avant-garde sociology and everyday life theory of Simmel, Benjamin, and others, who described the boredom, but also the mystery of industrial, bureaucratic modernity using aesthetic techniques learned from surrealism (Highmore, 2002); and the scientific humanism of Tarde, Freud, and others, who aimed to cultivate a scientific attitude among the general public and then to give voice to the masses without speaking for them – so without aggregating, classifying, or analysing, but instead by way of literary, aesthetic means: composition, depiction, and especially the juxtaposition of ‘luminous moments’ (Jardine, 2018). Many of the influences on Mass-Observation, however, were domestic or closer to home (Hubble, 2010; Jeffrey, 1978; MacClancy, 1995): developments in British social investigation and survey research (Booth, Rowntree, the New Fabian Research Bureau, the Pilgrim Trust, Political and Economic Planning), developments in market research (Gallup's British Institute of Public Opinion was also founded in 1937), people's fronts and popular alliances against the rise of fascism in Europe (expressed in the Left Book Club, Penguin Books, *Picture Post*, and the General Post Office or GPO Film Unit), and especially the disciplinary interests of its main founders.

Tom Harrisson was an anthropologist with interests in ethnography, participant observation, and the documenting of modern mass culture. Charles Madge had been a journalist for the *Daily Mirror*, was a member of the Communist Party, and, as a poet, was a member of a group of writers and artists based in Blackheath, London, interested in surrealism, psychoanalysis, the collective (un)consciousness of the nation, and the use of artistic forms – images, montages, juxtapositions – to map this (and, by doing so, to provide social therapy, emancipation, and social transformation). Humphrey Jennings, a third founder, was a writer, painter, photographer, film-maker, set-designer, and member of this Blackheath group. These different disciplinary interests have led some to suggest that Mass-Observation was a difficult coming together of Harrisson and Madge (and Jennings), anthropology and surrealism, the Worktown Project led by Harrisson in Bolton, and the National Panel led by Madge from Blackheath (e.g. Hinton, 2013; Jeffrey, 1978; MacClancy, 1995). There are certainly grounds for this interpretation and it wasn’t long before Jennings and Madge left Mass-Observation (in late 1937 and 1940, respectively), after which it became dominated by Harrisson. Still, the differences can also be overplayed. It is worth recalling that Harrisson and Madge met via the *New Statesman*, where a letter from Madge (the poet), introducing his idea of an ‘anthropology of our own people’, was printed on the same page as a poem by Harrisson (the anthropologist).

Whatever the precise intellectual distance between these individuals, the disciplinary multiplicity they represent constitutes one characteristic of Mass-Observation as a distinctive human science, at least in the late 1930s – before Madge left, before Harrisson focused increasingly on commissions for the Ministry of Information during the Second World War, and before the move to commercial market research that happened after the war (Sheridan, Street, and Bloome, 2000). Mass-Observation was an attempt to combine science and art, objectivity and subjectivity, rationalism and irrationalism – an experiment in ‘surrealist ethnography’ (Highmore, 2002) that presumably seemed possible at this experimental moment before academic professionalisation and the solidifying of intellectual boundaries during the post-war decades, and was likely made possible by a second characteristic of Mass-Observation's human science: empiricism. Different theoretical and methodological backgrounds did not matter so much in a project of radical empiricism where the collected material – observations, overheards, survey responses, directive responses – was meant largely to speak for itself (Jardine, 2018; Pocock, 1987). A third characteristic was Mass-Observation's accommodation of complexity, diversity, and ambiguity in public opinion (Kushner, 2004), at least compared to quantitative public opinion research of the time – the ‘skimpy statistics’ of ‘administrative sociology’ (Mass Observation, 1943) – and not least because of its experimentation with literary and aesthetic techniques of representation.

A fourth characteristic of Mass-Observation's human science was its prioritising of the ordinary: smoking, pub-going, wrestling, dancing, and so on (Madge and Harrisson, 1938, 1939); modern British culture understood as ‘mass’ culture and thought to be under-represented in both Parliament and the newspapers (Mass Observation, 1943). This was Mass-Observation as science *of the people*, documenting their beliefs, feelings, and behaviour. A fifth characteristic was the use of observers to collect and report observations (and their own thoughts and feelings). This was Mass-Observation as science *by the people*. A sixth characteristic was the publication of this material – edited and interpreted, but relatively lightly compared to most social research of the time (and since) – to multiple audiences: ministers and civil servants, who might use it to improve their understanding of the people, and so to improve planning and ultimately social conditions (Hinton, 2013; Madge and Harrisson, 1939); and citizens, who might use it to improve their understanding of society and their fellow citizens, thought necessary for democratic functioning (Madge and Harrisson, 1939), and to improve their understanding of themselves, to extend their consciousness, to bring themselves freedom, thought necessary for social transformation (Hinton, 2013; Hubble, 2010; Jennings and Madge, 1937; Pocock, 1987). Where the intended audience was ‘the people’, made up of mass observers and other ordinary people, this was Mass-Observation as *science for the people*.

In these last three characteristics, we see Mass-Observation as a democratic science of the people, by the people, for the people. In writing this, we’re inspired by MacClancy (1995: 495), who described Mass-Observation as ‘ethnography of the people by the people for the people’ (without reference to Abraham Lincoln's line from the Gettysburg Address: ‘government of the people, by the people, for the people’), and Pocock (1987: 416; emphasis in original), writing a few years before MacClancy, on how ‘the founders of the Mass-Observation “movement”, as it was called, all shared a belief that whether the observation was *of* the masses or *by* the masses (it was in fact a combination of both) it was certainly *for* the masses’. We’re also inspired by Highmore's (2002) interpretation of Mass-Observation as a political project concerned with democracy: against a background of rising fascism in Europe, Mass-Observation would confront elite representations of ‘the people’ with the ‘heterogeneous actuality’ of people.

Even in the late 1930s, Mass-Observation did not fully succeed in putting all characteristics of this distinctive science into practice. Its programme of publishing material to its observers and the broader public, for example, struggled to cope with too much collected material, too few editors (especially once the war took some of them away), and the high cost of early books like *May the Twelfth*, which put them beyond the reach of many ordinary people (Highmore, 2002; Pocock, 1987). Mass-Observation also became less distinctive over time as it responded to criticism of its approach, responded to the preferences and expectations of those in government and the private sector it depended on for commissions and income, and gradually tried to fit in more with an increasingly quantitative, statistical social science of public and market opinion (Hinton, 2013). Still, just like Mass-Observation was influenced by the human sciences of the late 19th and early 20th centuries, so Mass-Observation left its own effects on the human sciences of the mid and late 20th century. During the war, it influenced the Ministry of Information and inspired the establishment of its Home Intelligence Division, which took from Mass-Observation an interest in ‘morale’ – the mood or collective consciousness of the mass – and the possibility of shaping morale, governing it, through propaganda campaigns informed by research on the reception of such campaigns (Harrison, 2014; Highmore, 2017). Also during the war, through its mobilisation of ordinary people as observers and readers, it helped to embed and routinise social research in society (Savage, 2010); to create a new cadre and social identity of technically, intellectually, and scientifically engaged citizen-experts (ibid.); and to make opinion polling, mass surveillance, and self-observation a collective habit (Harrison, 2014). After the war, when Mass-Observation became increasingly dependent on commercial market research work, it modelled qualitative methods in that emerging field (Hinton, 2013). Then, from the 1950s and 1960s, the story of Mass-Observation functioned alongside Michael Young's Institute for Community Studies as an intellectual resource for the New Left, cultural studies, and others interested in working-class culture, everyday life, and its political effects (Campsie, 2016). Finally, from the 1960s and 1970s, materials collected by the original Mass-Observation became used by historians, initially to write social histories of the Second World War, then to write more broadly focused histories ‘from below’ (e.g. Addison, 1975; Calder, 1969; Hinton, 2010). These secondary uses of Mass-Observation sources were made possible by the founding of the MOA in 1975.

## The Mass Observation Archive and the human sciences

The MOA opened as an archive of papers from the original Mass-Observation with Tom Harrisson as its first director. Within a few years, however, under its second and third directors – David Pocock (1976–90) and Dorothy Sheridan (1990–2008) – it became an active research organisation in its own right. Today, its ongoing research projects include the Mass Observation Project (MOP), established in 1981 when it was known as ‘Mass-Observation in the 1980s’ or ‘The Inflation Project’ (because life in Britain at the beginning of that decade was characterised by high inflation, among other things). The MOP sends directives to a panel of volunteer writers three times a year and so might be seen as the revival of the National Panel run by the original Mass-Observation, though with some differences that we’ll come to shortly. Ongoing projects also include the 12 May project, established in 2010, which collects day diaries from across the UK every year on 12 May and so might be seen as the revival of the day diaries collected by the original Mass-Observation and some of the broader research ideas behind Jennings and Madge's (1937) *May the Twelfth*. These projects and others, and the ongoing archiving and publishing work done by the MOA, constitute what might be thought of as a contemporary Mass Observation centred on the MOA and both connected to and distinct from the original Mass-Observation. Furthermore, this contemporary Mass Observation has now been in existence for almost half a century. Scholars began taking the human science of the original Mass-Observation seriously during the 1970s (e.g. Jeffrey, 1978), just four decades after its first years of work. It is time to take the contemporary Mass Observation seriously as its own form of human science with its own place in the history of the human sciences, building on the work of others (e.g. Casey, Courage, and Hubble, 2014; Hinton, 2016, 2021; Pollen, 2013; Sheridan, 1993, 1996, 2021; Sheridan, Street, and Bloome, 2000).

If the original Mass-Observation influenced the contemporary Mass Observation, then so did other human sciences of the 20th century. One was anthropology, but less the anthropology of Harrisson, founder of the original Mass-Observation and first director of the MOA, whose anthropology aspired to positivist scientific rigour (Sheridan, Street, and Bloome, 2000), and more the anthropology of his successor as director, David Pocock, founder of the MOP. Pocock's anthropology was the ‘Oxford’ or ‘social’ anthropology associated with Evans-Pritchard (ibid.). It was a humanistic anthropology concerned with how meaning is constructed and interpreted in everyday life, and people's own concepts, models, and theories of social life. Pocock described his own version of this as ‘personal anthropology’: the study of socially constructed ideas, concepts, and theories of self and society that are held by people and used to inform practices and relationships. The MOP would be used to access these ‘personal epistemologies’ (ibid.).

After Pocock, the next director of the MOA also left her mark on the MOP. Dorothy Sheridan describes herself as a feminist interested in women and gender, and in combining social documentary and autobiography, the public and the private, the political and the personal (Sheridan, 2021). She led the MOP for almost two decades, influenced by this feminism and associated interests in life story projects, community writing, and ethnographic and participatory research (Sheridan, 1996). These interests led her to view the MOP as primarily a life history project, as opposed to a form of social survey research (ibid.). In this view, the MOA becomes primarily an archive of autobiographical writing, produced from Sheridan's directives on childhood, education, work, marriage, ageing, and so on, from which autobiographical essays might be produced by researchers – influenced by the traditions already mentioned and also numerous allied movements in history: social history, history from below, the History Workshop movement, oral history, and the biographical turn in history (Hinton, 2016).

In the previous two paragraphs, we can already see the bones of the contemporary Mass Observation's human science. It is a project informed by social anthropology and feminism that collects autobiographical writing and, through that, provides access to people's understandings of self and society – especially the perspectives of women (who’ve been consistently over-represented on the MOP panel; not by design, but for many reasons, including that women have been more attracted to a project informed by feminism, even when that influence has not been foregrounded; Sheridan, Street, and Bloome, 2000). Let us now add flesh to these bones by identifying some additional characteristics of this human science, including characteristics that situate it in relation to the original Mass-Observation. Like the original Mass-Observation, research centred on the MOA focuses on the everyday lives of ordinary people. Indeed, writers for the MOP mostly identify as ‘ordinary people’, by which they mean people without success, fame, or influence, who don’t usually get an opportunity to go on record, unlike politicians, celebrities, journalists, or academics (Sheridan, 1996). The difference is that while the National Panel of the 1930s and 1940s contained many young men – and was skewed towards lower-middle-class teachers, librarians, secretaries, clerks, and shopkeepers, often on the political left (Sheridan, Street, and Bloome, 2000) – the panel of the MOP has been consistently skewed towards older women (ibid.). So one characteristic of the contemporary Mass Observation is its particular focus on the lives and experiences of women.

Another characteristic is that archivists at the MOA mobilise ordinary people, especially ordinary women, as writers – encouraging and enabling them to ‘write themselves’ and, by doing so, ‘to write Britain’ (Sheridan, Street, and Bloome, 2000). However, compared to the original Mass-Observation, there has been less mobilisation of ordinary people through publication of this writing. Recall that the original Mass-Observation sought to mobilise people as good democratic subjects, or even revolutionary subjects, by two means: recruiting them as observers, and publishing observations back to them as readers. The MOA has been more active in publishing collections of material from the 1930s and 1940s (e.g. Calder and Sheridan, 1984; Garfield, 2005; Sheridan, 2009) than from the MOP or 12 May project. For these reasons, it is tempting to see the original Mass-Observation as oriented towards the present, collecting material for use by researchers, government, and citizens at the time – though much of that material was only ever used by historians at a later date – and to see the contemporary Mass Observation as oriented towards the past and future: an archive publishing material from history and collecting material for use by future historians. Indeed, this is how the contemporary Mass Observation is often viewed by publishers and journalists, perhaps because it is centred on the MOA, an archive known by many for its Second World War collections. This is also how many writers for the MOP view their contributions: as the voices of ordinary people, speaking to future historians (Kramer, 2014; Sheridan, Street, and Bloome, 2000).

However, the temptation to view the contemporary Mass Observation as oriented towards the past and the future, as opposed to the present, should be resisted. MOP directives are often commissioned by social scientists focused on the present. The archivists frequently update contributors on how their contributions are being used in current research. It is better to view the contemporary Mass Observation as concerned more with collecting writing (to be read by researchers) than publishing writing back (to correspondents); as concerned with mobilising correspondents more as writers (and observers) than as readers (of Mass Observation's collections); as concerned more with the first two parts of the original Mass-Observation's democratic science (of the people, by the people, for the people). Even then, some caveats are due. There have been projects aimed at bringing writing for the MOP to broader audiences, such as the JISC-funded ‘Observing the 1980s’ project (a collaboration with the British Library; see https://blogs.sussex.ac.uk/observingthe80s/). At the time of writing, there are ongoing projects that could do something similar for more recent material, such as ‘Mass Observing COVID-19’ (funded by the Wellcome Trust). The activities of the contemporary Mass Observation are also constrained by practical considerations, with much of the MOA's current funding dependent on publishing of material behind the paywall of Adam Matthew Digital.

Returning to the contemporary Mass Observation's distinctive human science, a third characteristic of this science is the way it generates and archives writing that is both subjective, with panellists writing to construct and promote their identity (Nettleton and Uprichard, 2011; Sheridan, Street, and Bloome, 2000), and ‘intersubjective’ (Pollen, 2014) or ‘dialogic’ (Bloome, Sheridan, and Street, 1993; Salter, 2010). Panellists write in response to directives not just as respondents, but as ‘correspondents’ engaged in an ongoing conversation with the archivists, sometimes over years and even decades (Sheridan, Street, and Bloome, 2000). These long-term, trusting relationships encourage particularly ‘frank’ writing, while producing longitudinal sets of writing that capture uncertainties, tensions, and contradictions (Wilson-Kovaks, 2014) – the ambiguities, complexities, and confusions that make up human experience in full (Hinton, 2016). As correspondents, panellists also regularly contest and seek to transform the position they have been given, whether in the directives themselves, in broader public discourses, or in more private interactions (Sheridan, Street, and Bloome, 2000). The materials archived by the MOA, therefore, lend themselves especially well to researching top-down and bottom-up processes. In these materials, researchers have identified the ‘cultural worlds’ or ‘worlds of discourse’ in which writers are situated, and how people construct from these worlds their own distinctive selfhoods, driving historical processes as historical agents working on received cultural norms (Hinton, 2010). Researchers have identified the standards and codes circulating in society, how people receive them, their dis/comfort with them, and what people do with them (Langhamer, 2016). Researchers have identified the sociological constructs available to people as resources, but also people's lay articulations of those constructs – their practices of selection, interpretation, appropriation, incorporation, and contextualisation (Wilson-Kovaks, 2014).

A final characteristic of the MOA and its research projects and materials is their location between the humanities and social sciences, and the way they draw from and attract sociologists, human geographers, literacy scholars, historians, creative practitioners, and many others. This brings us to how the contemporary Mass Observation has influenced – or, better, the function it has performed for – the human sciences of the 21st century. In the last couple of decades, the MOA has generated and hosted methodological debates between scholars from across the arts, humanities, and social sciences regarding how to interpret written sources and analyse qualitative data (see Casey, Courage, and Hubble, 2014; Pollen, 2013). One example is the debate on how representative the MOP correspondents are when compared to wider British society. This debate has clarified numerous potential responses to the more broadly relevant question: how should researchers working with diaries and similar materials confront the challenge of representativeness?

One response from social scientists working on data collected by the MOP has been to challenge claims the panel is skewed. Such claims are often made on the basis of metadata, like the occupations registered by panellists at the time of joining, which have led to claims the panel is skewed towards the middle classes. Casey (2020) rejects such claims, arguing that metadata of this kind is of limited value for such purposes. While panellists appear to be middle-class, given their occupations at a particular point in time, many identify as working-class on the basis of their social biographies and past social mobility – something evident in their writing, but not in the metadata. A second response, also from social scientists, has been to accept claims the panel is skewed and focus research accordingly. So researchers have used MOP data to make claims about women, who, as we’ve seen, have been over-represented on the panel, and who can be given a voice using MOP data when often the voices of women are marginalised in public discourse (Baker and Geringer, 2018). Researchers have also used MOP data to make claims about other groups over-represented on the panel: the educated middle class (Savage, 2007); the upwardly mobile (Casey, 2020); particularly engaged, dutiful citizens (Manning, 2018); volunteers, who tend to be female and older (Lindsey and Bulloch, 2014); genealogists, who tend to be female, older, and middle-class (Kramer, 2011); and gardeners, who tend to be female, older, middle-class, and white – so just like many of the MOP panellists (Bhatti *et al*., 2009). A third response from social scientists has been to correct the skew by sampling within the panel, filling quotas for age, gender, occupation, and place of residence, to achieve broad social coverage and descriptive saturation (Clarke *et al*., 2018; May, 2018; Salter, 2010).

If these responses have been provided by social scientists, then a further set of responses have been provided by scholars approaching Mass Observation from the arts and humanities. This latter set of responses has not so much engaged with the problem of representativeness on its own terms – rejecting claims of a skew, working with the skew, or correcting for the skew – as challenged those very terms. So a fourth response treats MOP writers not as ‘representative cases’ who provide access to views prevalent in the social groups they represent, but as ‘telling cases’ who provide access to something more ‘essential’ (Bloome, Sheridan, and Street, 1993). Here, Bloome, Sheridan, and Street draw on Clyde Mitchell, who in turn drew on Florian Znaniecki. We might say that such a response rejects enumerative induction, or the generalising from many cases with the same characteristics, for analytical induction, or what Morgan (2021) terms ‘thinking with cases’: the abstracting of essential findings from one or a few cases, which are then expected to be relevant in other cases. A fifth response treats MOP writers as cultural actors. They act by engaging with cultural resources: categories, storylines, subject positions, folk theories. Here, writing in the MOA provides access to the cultural resources circulating in society at a particular historical moment, and how people actively use and remake those cultural resources (Clarke *et al*., 2018; Gazeley and Langhamer, 2012; Nettleton and Uprichard, 2011; Salter, 2010; Savage, 2007).

A final response sees the problem of representativeness and raises the problem of representation. If the former problem is a sampling problem – a statistical problem at the data collection or ‘input’ stage of research (of particular interest to social scientists) – then the latter problem is an aesthetic problem: a literary problem at the ‘output’ or ‘writing up’ stage of research (of particular interest to arts and humanities scholars). This response draws lessons from the original Mass-Observation. Influenced by surrealism, Madge and Jennings approached the challenge of representing everyday life in the late 1930s as primarily an aesthetic challenge – a challenge of composition and depiction – to be addressed by aesthetic techniques: image, close-up, panorama, juxtaposition, montage, collage (Highmore, 2002; Hubble, 2010; Jardine, 2018; Marcus, 2001). In this final response, we see clear differences between the concerns of differently trained and interested researchers when approaching the MOA. Across all six responses to the question of representativeness, we see how the contemporary Mass Observation, by staging methodological debates between scholars from across the arts, humanities, and social sciences, has contributed to, or facilitated contributions to, development of the human sciences in the 21st century.

## Middletown and the human sciences

One influence of the contemporary Mass Observation has been on the EDLM project, the basis for the fourth article in this Special Section. But to situate the EDLM project in relation to Mass Observation, and in the human sciences more broadly, we need to revisit the early 20th century and the first Middletown study, which influenced the original Mass-Observation and a series of subsequent Middletown studies, including the EDLM project.

The first Middletown study, completed by Robert S. Lynd and Helen Merrell Lynd in the mid 1920s and published as *Middletown* in 1929, was a ‘field investigation’ aiming ‘to study synchronously the interwoven trends that are the life of a small American city’ (Lynd and Lynd, 1929: 3) and so ‘to reveal interrelations in the maze of interlocked, often contradictory, institutional habits’ (ibid.: 497). By focusing on ‘life’ in general, at least in a ‘small American city’, collecting data about everyone in that city (or not quite everyone, as we shall see), and circulating those data widely (by way of a book that became a surprise bestseller), the study departed from other sociological research of the time. Whereas the urban sociology of the Chicago School, for example, studied social problems, deviation from the norm, and ‘deviants’ – with a view to solving social problems, governing deviant groups, and guiding social reform – the Middletown study pursued self-understanding on behalf of the American nation (Cacamo, 2000; Igo, 2008).

The Lynds chose Muncie, Indiana as their field site for both its typicality and its atypicality. On the one hand, Muncie exhibited ‘many features common to a wide group of communities’ (Lynd and Lynd, 1929: 3), including a temperate climate, a rapid rate of growth, an industrial culture, a substantial and largely self-contained artistic life, an absence of acute local problems, and a location in the Middle West. In this sense, the Lynds viewed Muncie as ‘Middletown’. On the other hand, Muncie was chosen for its particularities. It was a city ‘compact and homogeneous enough to be manageable in such a total-situation study’ (ibid.: 7). It was chosen for its population size (under 50,000), because it was ‘self-contained’, and for its ‘small Negro and foreign-born population’ (ibid.: 8) – which the study ignored, confining its focus to ‘native whites’ (ibid.: 9). This focus was determined by the research design. The Lynds were interested in cultural change and the relationship between ‘constant native American stock and its changing environment’ (ibid.: 8). They viewed racial change as a ‘complicating factor’ to be controlled for, which they believed could be achieved in ‘homogeneous’ Muncie. The treatment of racial change in the study, or the lack thereof, would provide one target for criticism in the large secondary literature stimulated by this first Middletown study (e.g. Cacamo, 2000; Igo, 2008; Jensen, 1979).

Focusing on Muncie's ‘native whites’, but otherwise keen to study ‘life’ in the city, the Lynds constructed their approach from resources provided by functionalist cultural anthropology. Six areas of human activity were identified for investigation: getting a living, making a home, training the young, using leisure, engaging in religious practices, and engaging in community activities. The primary technique was ‘participation in local life’ – in church services, school assemblies, court sessions, political rallies, civic luncheon clubs, and so on – though other techniques included ‘examination of documentary material’, ‘compilation of statistics’, ‘interviews’, and ‘questionnaires’. If the approach and techniques were influenced by anthropology, interpretation was influenced by Thorstein Veblen. *The Theory of the Leisure Class* had been published in 1899 and had characterised the new American industrial aristocracy by their ‘pecuniary values’ (Veblen, 2007[1899]). The Lynds concluded that such ‘pecuniary considerations’ now dominated both business-class and working-class life in Muncie, making Middletown a ‘pecuniary community’ (Lynd and Lynd, 1929: 502).

On publication, *Middletown* sold well and received much attention. It was at the same time both a morality tale of decline from Gemeinschaft to Gesellschaft, and a reassuring depiction of American society that hid most of its harshest criticisms in footnotes (Jensen, 1979). It was viewed as objective and scientific compared to previous studies, and as relevant to both Muncie and America as a whole – at a time when there was an appetite for studies of American society, which was assumed to be changing, industrialising, urbanising, nationalising, modernising (Igo, 2008). The Lynds followed *Middletown* with *Middletown in Transition* (1937), a re-study of Muncie during the boom years of the late 1920s and the bust years of the early 1930s. The legacy of these two studies was modest in methodological terms. They inspired community studies like those completed by W. Lloyd Warner during the 1940s, but such ‘middle-range’ research became eclipsed in mid-20th-century American sociology by ‘macroscopic theoretical studies’ and ‘microscopic technical studies’ (Jensen, 1979). In other ways, however, their legacy was substantial. For Sarah Igo (2008), they were two of a number of studies completed in America during this period – including the public opinion polls of George Gallup and Elmo Roper, and the sexual behaviour reports of Alfred Kinsey – that disclosed America to itself as an emerging national society, but also located a midpoint in that society, a typical America, an average America, which informed the self-understanding of Americans, their social imaginations and identities, and shaped America's public sphere around ‘the averaged American’, as opposed to America's minorities.

Another legacy of these original Middletown studies was that Muncie became seen by social scientists, market researchers, and journalists as a bellwether or barometer for modern American society, such that it has been studied and restudied frequently over the last century, perhaps more than any other American community (Connolly, 2005). Other notable Middletown studies include Middletown III and IV, led by Theodore Caplow in the late 1970s (1977–9) and at intervals thereafter (1989 and 1999), and published in many journal articles and books, including *Middletown Families* (Caplow, Chadwick, and Bahr, 1982) and *All Faithful People* (Caplow, Bahr, and Chadwick, 1983). These studies were both similar and different to the original studies (Cacamo, 2000). They were similar because Caplow had been a student of Robert Lynd and sought to repeat Lynd's original questions and categories as faithfully as possible. They were different because Caplow and his team were sociologists trained during the mid 20th century who preferred social survey research to participant observation. Another notable Middletown study was led by Luke Eric Lassiter around the turn of the 21st century and published as *The Other Side of Middletown* (Lassiter *et al*., 2004). As an anthropologist, Lassiter preferred participant observation to social survey research, but distinguished his project from the original Middletown studies in other ways. Its purpose was to address the lack of ‘African American history and experience’ in the original studies (and many of the restudies they inspired). To this end, it was designed as a collaborative ethnography between Lassiter, his students, community leader Hurley Goodall, and other community members. It sought to practice an engaged social science, to set up dialogues, and to change the views of participants (at least some of whom reported changed views from participating in the study; Lassiter, 2012).

Where does the EDLM project fit into this field? It began in 2016 as an undergraduate seminar led by Patrick Collier at the Virginia B. Ball Centre for Collaborative Inquiry. It is currently a project of the Centre for Middletown Studies, directed by James Connolly. In its current location, it might be seen as the latest in a long line of Middletown studies. However, its human science is closer to some of those studies than others. As a collaboration between Ball State University faculty and the citizens of Muncie, it is closer to the collaborative, engaged study of Lassiter and colleagues than, say, the Middletown III and IV studies. The other thing to note about the EDLM project, by way of introduction, is that its design and practice have been influenced by Middletown studies (both original and more recent), but also Mass Observation (both original and contemporary). The EDLM project runs a panel of volunteer writers who keep diaries of their daily lives in a way similar to the National Panel of the original Mass-Observation and the MOP of the contemporary Mass Observation. These diaries are archived in a way similar to how writing for Mass Observation is archived by the MOA, but they are also published online as a ‘digital commons’ where people are encouraged to gather, read, comment, and increase their awareness of their own everyday life and the lives of others – in a way similar to how observations were published to observers by the original Mass-Observation as part of its democratic science of the people, by the people, for the people.

## Processing the COVID-19 pandemic

For the best part of a century, Mass Observation and Middletown have provoked conversations between scholars from across the human sciences. In this Special Section, we add another conversation to the mix. We present articles from sociology (Lyon and Coleman), human geography (Clarke and Barnett), visual and material culture (Pollen), and English and history (Collier and Connolly). These articles represent four engagements with diaries and other forms collected by the MOA and EDLM project during the COVID-19 pandemic. Together, they constitute an engagement with archiving the pandemic in two senses: the archiving of diaries, directive responses, and images by archives like the MOA; and the archiving of research on these diaries and other forms – the initial engagements between researchers and these materials – by *History of the Human Sciences*. We have seen how Mass Observation has been oriented towards the present (collecting material for use by researchers, government, and citizens at the time) and the future (collecting material for use by future historians). This Special Section is similarly oriented. It is an attempt both *to know the pandemic* from the human sciences in the present and *to archive knowing the pandemic* from the human sciences for the future.

In the first article, Lyon and Coleman discuss responses to the Summer 2020 Mass Observation directive they commissioned on ‘COVID-19 and time’. Inspired by the rhythmanalysis of Lefebvre and Régulier, their analysis focuses on rhythm, arrhythmia, eurhythmia, and related concepts and phenomena. We learn how the pandemic ruptured everyday rhythms. It was a temporal shock. It stopped, halted, or froze time. Activities and plans were cancelled. The strange present of the pandemic was detached from the familiar pre-pandemic past and the now uncertain future. The movements that usually punctuate the day were lost. There was the shock of absent rhythm, but also the challenge of new rhythms, often related to home schooling, working from home, and interactions between the two. With everyday rhythms having been ruptured, time was experienced as blurred, merged, repetitive, monotonous, dull, drifting, suspended, undifferentiated, lacking punctuation points, lacking shape, lacking distinction (between day and night, or different days or weeks). In this context, there was a heightened desire for rhythm. People remade rhythms using devices (calendars, diaries, clocks, alarms, lists) and practices (watching the daily government briefings, taking daily walks, scheduling Zoom coffee breaks or family mealtimes, attuning to nature). By disrupting the rhythms of everyday life, the pandemic showed up the centrality of rhythms to everyday life.

The second article also draws on responses to this Summer 2020 directive, alongside responses to Mass Observation's Spring and Special 2020 directives, and a sample of the diaries collected by Mass Observation on 12 May 2020 (i.e. Mass Observation's COVID-19 collections from the first eight months of the pandemic). Clarke and Barnett use these collections to study how governing the pandemic through scientific literacy – by trying to get ordinary people to see like epidemiologists – played out. How did people use the statistics, charts, maps, identities, and roles – ‘the science’ – provided to them? Did they come to think in terms of populations, groups, rates, trends, distributions, risks, and capacities? Did they become epidemiological publics with epidemiological imaginations? Did they develop their own lay epidemiology that ‘spoke back’ – resisted, complained about, reinterpreted the guidance of – public health authorities? From this article, we learn how there were many opportunities for people to engage with ‘the science’. This engagement was generally with science *plural*. For many people, such engagement led to seemingly confident and comfortable use of epidemiological terms and concepts. However, engagement with some of the subject positions offered by epidemiology – especially ‘vulnerable’ and ‘at risk’ – tended to be less comfortable, with subject positions sometimes refused, sometimes with consequences for compliance with government regulations and advice. Furthermore, many people used their scientific literacy in a way perhaps not intended or anticipated by governments: to compare and judge governmental performance, and so to hold governments to account.

Our third article also draws on Mass Observation's COVID-19 collections from the first period of the pandemic – both directive responses and day diaries – but focuses in particular on submissions including photographs, drawings, paintings, written visual descriptions, and writing about COVID-19's image cultures. Pollen is interested in how the pandemic was visualised, pictured, seen. She notes how the images and words of correspondents with Mass Observation were situated in a wider visual culture comprised of stylised renderings of the spiked, spherical virus (the invisible main actor); photographic documentation of the stage (empty streets, social distancing signage, etc.) and other actors (heroes, such as front-line health workers in protective equipment; villains, such as those breaking social distancing rules); and participatory crowdsourced public photographic collecting projects (aiming to create connections between ‘stay home’ communities, to speak about shared experiences, to record the historical moment, and to externalise the emotions precipitated by the new conditions – and often making explicit reference to the original Mass-Observation as their inspiration). In Mass Observation's COVID-19 collections, Pollen found numerous images: scenes from nature, barricaded playgrounds, public health signage, the pets and gardens people took comfort in, the art and craft projects people spent time on, the bread people baked, the meals that became so central to people's days (see also Lyon and Coleman). Pollen also found plenty of visually inflected, image-rich, imaginative writing. The pandemic encouraged people to see everyday life as even more strange and worthy of scrutiny than usual. It produced fresh perceptions, heightened senses, vivid dreams. People wrote about their dreams, fantasies, and mental images; watching and being watched (the new surveillance); feeling like they were in a painting, photograph, or film; and feeling encouraged to look more carefully at their worlds.

In the final article, Collier and Connolly take us from the UK to the USA (Muncie, Indiana), and from Mass Observation to the EDLM project. This article also focuses on time and rhythm, bringing us back to Lyon and Coleman's article. Collier and Connolly are interested in how the pandemic distorted experiences of time, but also how this was influenced by place – because place, or local structures of feeling, curates the stories available to people. They ask: how do experiences of time and place inflect, or produce variations of and problems for, the autobiographical selves we construct? We learn that during the pandemic, people in Muncie experienced time in the present as confusing and disorientating. Routines were altered (e.g. by working from home). Daily rhythms were scrambled, producing arrhythmia. Durations were also scrambled, with short periods feeling long and long periods feeling short. People experienced an inability to plan for the midterm future and anxiety about the long-term future. Whereas Muncie usually shapes the writing of participants in the EDLM project, including their orientation to the future, giving them cultural scripts against which to orient themselves as individuals, the pandemic seems to have changed this. People wrote less about the city, industrial decline, spluttering recovery, class conflict, provincialism. They wrote more about themselves, their families, their houses. Or, jumping the local scale, they wrote more about the nation and the globe.

Let us finish with some concluding points generated by reading across the four articles. Collections in the MOA have often been used to study top-down and bottom-up processes, not least because of the intersubjective writing Mass Observation encourages from correspondents. It would appear that Mass Observation's COVID-19 collections can be used in such a way. Clarke and Barnett demonstrate this, finding in the collections evidence of positioning: how people exercise choice in relation to categories, storylines, and subject positions; how they position themselves using the subject positions made available to them; and how they recognise themselves as members of some categories (but not others), and accept certain subject positions made available to them (but not others). The contemporary Mass Observation mobilised people to write about everyday life during the COVID-19 pandemic and especially to write dialogically about authority (e.g. government regulations and advice), standards (e.g. of handwashing or social distancing), and subject positions (e.g. the family member with caring responsibilities, or the furloughed worker with time for new hobbies).

Perhaps the most common way that materials in the MOA have been approached is as life history or autobiographical writing. This is especially the case for responses to directives of the MOP. It is also the case for submissions to the EDLM project. In both cases, the writing prompted by the COVID-19 pandemic appears to have challenged such an approach. Lyon and Coleman found that responses to the Summer 2020 MOP directive contained little autobiographical writing. They wonder if life writing was just too difficult for people during the pandemic. Instead, these responses contained ‘small stories’ and ‘fragments’ of a ‘live present’. They show people grappling with the pandemic as it unfolded – and were a means of such grappling too. Similarly, Collier and Connolly found that participants in the EDLM project, who usually would write about the life-time, looking back and forwards as they did so, wrote about other things instead. The pandemic obscured participants’ connection to the future. It undermined their sense of time as continuous, and so their sense of belonging. It disrupted, deferred, or reshaped their life narratives, and so exerted pressure on their self-understanding and self-presentation.

This absence of life writing in the COVID-19 collections is also noted by Pollen, who approaches images and text in the MOA as ‘real-time processing’ of the pandemic and the experiences and questions it generated. Correspondents seemed to be asking themselves: what is this time we are living through? How can we make sense of it? Pollen also reminds us that Mass Observation correspondents are best seen as not only producers of research data for others to analyse, but also researchers or analysts – or processors – in their own right. In turn, we are reminded that Mass Observation encourages a democratic science *of* the people, *by* the people. The original Mass-Observation also encouraged a democratic science *for* the people. We have seen how this last function is no longer a priority for the contemporary Mass Observation, but it remains a priority for – is given a second life by – the EDLM project. Collier and Connolly describe how the EDLM project aims to build and model community connection and dialogue in Muncie. It attempts to reinvigorate Muncie's public sphere by constructing *and sharing* a digital archive – to be shared with and read by the volunteer writers themselves. There are continuities and discontinuities, then, between the projects and collections discussed in this Special Section. One function the four articles perform together, though, is to archive the initial processing by researchers of materials in the COVID-19 collections, which in turn contain the real-time processing by ordinary people of the pandemic.

Finally, if these COVID-19 collections demand new approaches, beyond previously dominant life history approaches, they also demand new or additional methods. Pollen argues that Mass Observation's collections increasingly require visual methods. These have long been present in Mass Observation. The original Mass-Observation foregrounded ‘observation’, employed painters and photographers, and sought poetic images of mass society. Pollen notes this presence, but also the marginal status of visual methods in Mass Observation. The original Mass-Observation used painters and photographers for limited objective observation, and sought poetic images largely in writing, while the contemporary Mass Observation, especially the MOP, is focused primarily on autobiographical writing. The point here is relevant to Mass Observation and perhaps the EDLM project, and especially their COVID-19 collections, but also more broadly. Visual methods, Pollen suggests, are particularly appropriate to materials generated by the pandemic – whether archived by Mass Observation or not. The pandemic heightened the senses, generating imaginative, especially image-rich materials. More broadly still, such methods are increasingly appropriate to new collections of mass processing. As Pollen notes, technological developments have changed the character of submissions to projects archiving everyday life. They increasingly include photographs, screengrabs, and other visual forms. Given these changes – to submissions, methods, and approaches – Mass Observation and Middletown should be viewed as both objects of knowledge for historians of the human sciences in the 20th century, and living human sciences in the 21st century.
